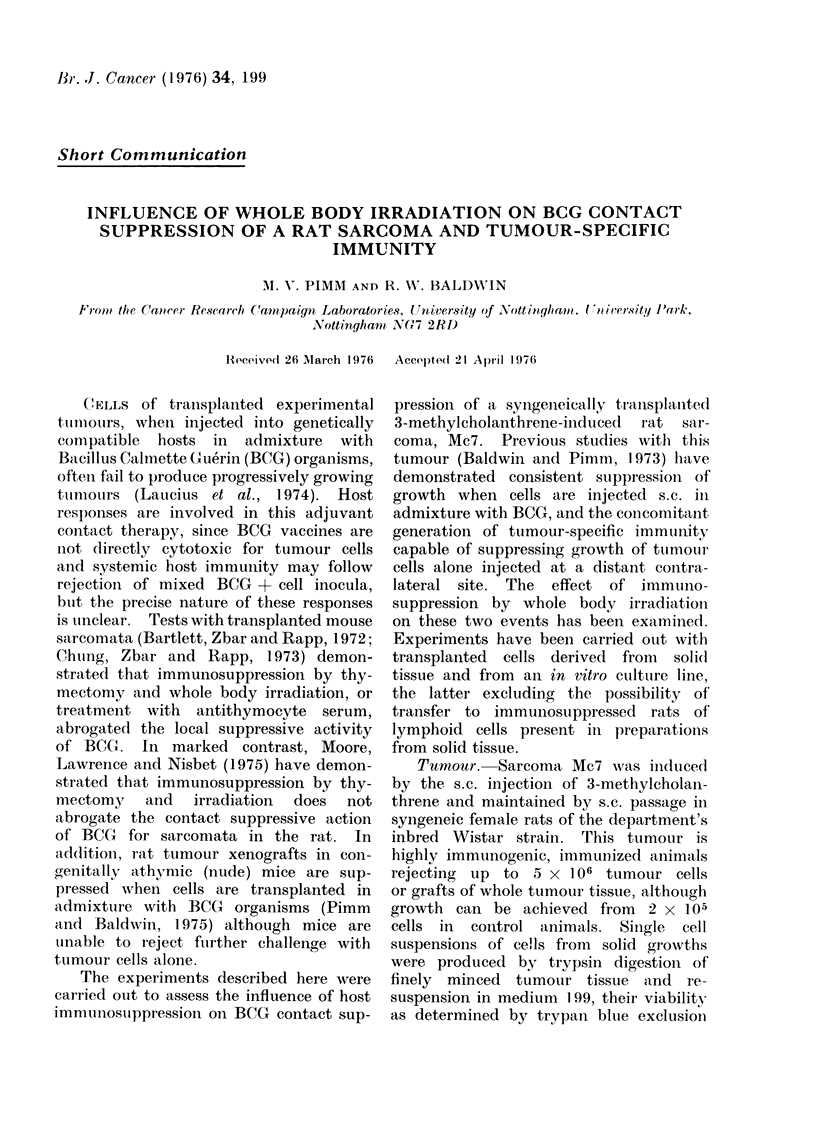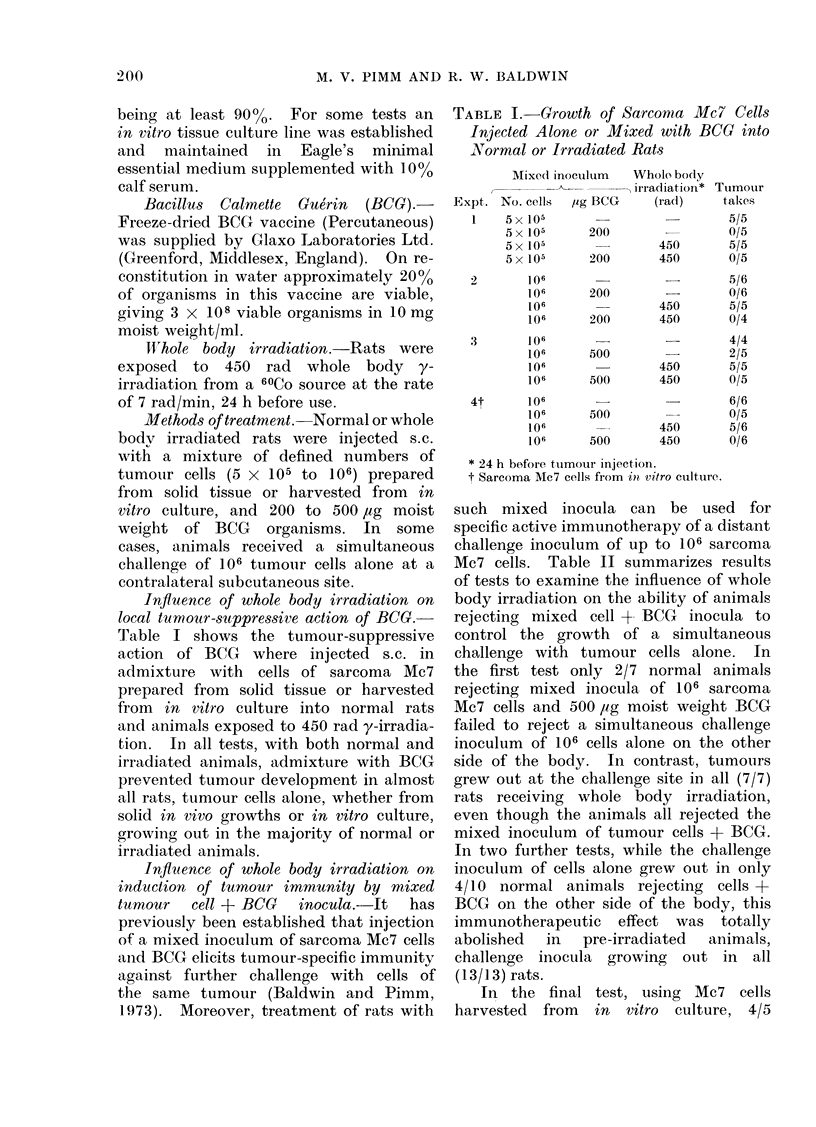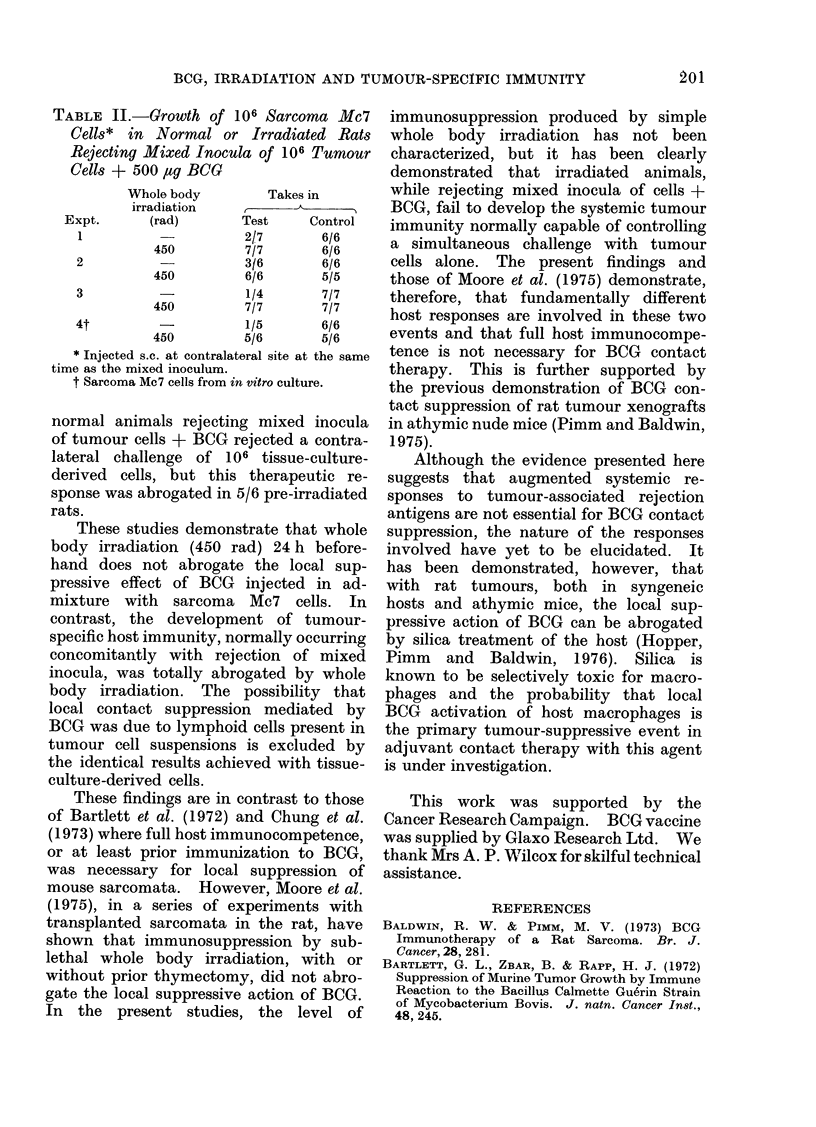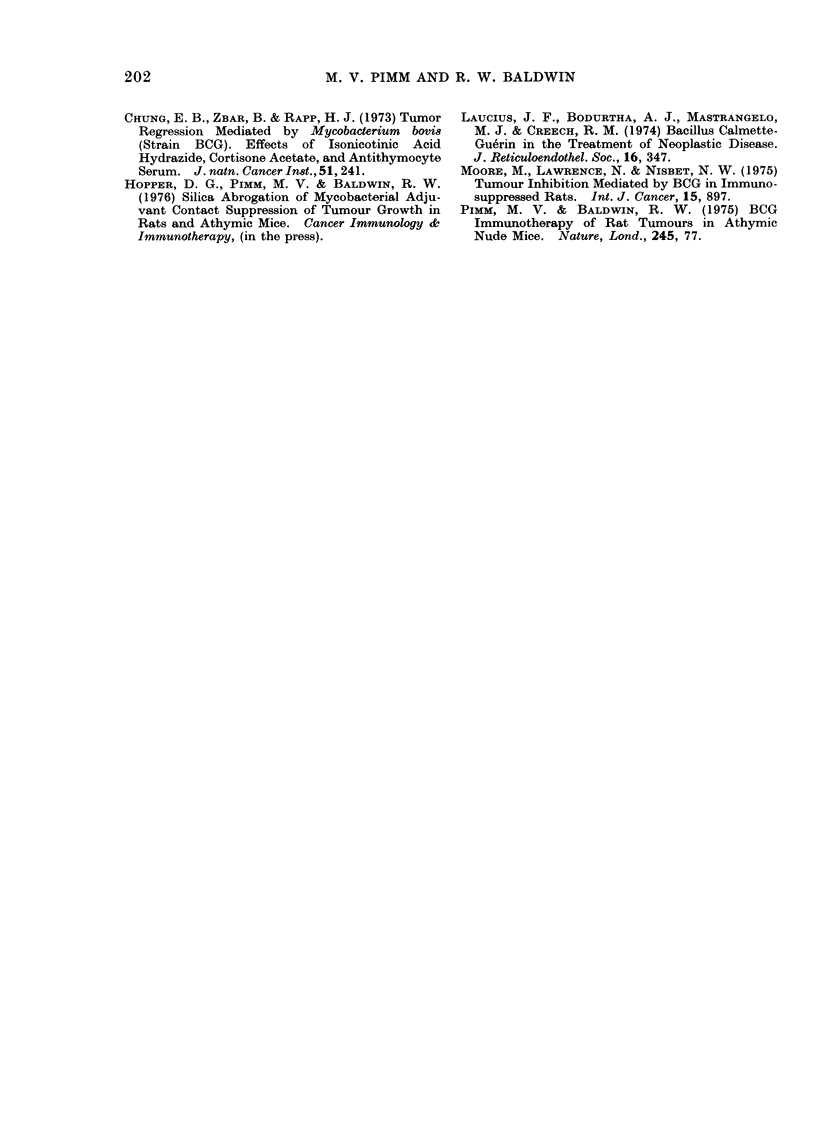# Influence of whole body irradiation on bcg contact suppression of a rat sarcoma and tumour-specific immunity.

**DOI:** 10.1038/bjc.1976.143

**Published:** 1976-08

**Authors:** M. V. Pimm, R. W. Baldwin


*Br. J. Cancer* (1976) **34**, 199

***Short Communication***

### INFLUENCE OF WHOLE BODY IRRADIATION ON BCG CONTACT SUPPRESSION OF A RAT SARCOMA AND TUMOUR-SPECIFIC IMMUNITY

M. V. PIMM AND R. W. BALDWIN

*From the Cancer Research Campaign Laboratories, University of Nottingham, University Park, Nottingham NG7 2RD*

Received 26 March 1976 Accepted 21 April 1976

CELLS of transplanted experimental tumours, when injected into genetically compatible hosts in admixture with Bacillus Calmette Guérin (BCG) organisms, often fail to produce progressively growing tumours (Laucius *et al.*, 1974). Host responses are involved in this adjuvant contact therapy, since BCG vaccines are not directly cytotoxic for tumour cells and systemic host immunity may follow rejection of mixed BCG + cell inocula, but the precise nature of these responses is unclear. Tests with transplanted mouse sarcomata (Bartlett, Zbar and Rapp, 1972; Chung, Zbar and Rapp, 1973) demonstrated that immunosuppression by thymectomy and whole body irradiation, or treatment with antithymocyte serum, abrogated the local suppressive activity of BCG. In marked contrast, Moore, Lawrence and Nisbet (1975) have demonstrated that immunosuppression by thymectomy and irradiation does not abrogate the contact suppressive action of BCG for sarcomata in the rat. In addition, rat tumour xenografts in congenitally athymic (nude) mice are suppressed when cells are transplanted in admixture with BCG organisms (Pimm and Baldwin, 1975) although mice are unable to reject further challenge with tumour cells alone.

The experiments described here were carried out to assess the influence of host immunosuppression on BCG contact suppression of a syngeneically transplanted 3-methylcholanthrene-induced rat sarcoma, Mc7. Previous studies with this tumour (Baldwin and Pimm, 1973) have demonstrated consistent suppression of growth when cells are injected s.c. in admixture with BCG, and the concomitant generation of tumour-specific immunity capable of suppressing growth of tumour cells alone injected at a distant contralateral site. The effect of immunosuppression by whole body irradiation on these two events has been examined. Experiments have been carried out with transplanted cells derived from solid tissue and from an *in vitro* culture line, the latter excluding the possibility of transfer to immunosuppressed rats of lymphoid cells present in preparations from solid tissue.

*Tumour.*—Sarcoma Mc7 was induced by the s.c. injection of 3-methylcholanthrene and maintained by s.c. passage in syngeneic female rats of the department's inbred Wistar strain. This tumour is highly immunogenic, immunized animals rejecting up to $5 \times 10^6$ tumour cells or grafts of whole tumour tissue, although growth can be achieved from $2 \times 10^5$ cells in control animals. Single cell suspensions of cells from solid growths were produced by trypsin digestion of finely minced tumour tissue and resuspension in medium 199, their viability as determined by trypan blue exclusion being at least 90%. For some tests an *in vitro* tissue culture line was established and maintained in Eagle's minimal essential medium supplemented with 10% calf serum.

*Bacillus Calmette Guerin (BCG)*.—Freeze-dried BCG vaccine (Percutaneous) was supplied by Glaxo Laboratories Ltd. (Greenford, Middlesex, England). On reconstitution in water approximately 20% of organisms in this vaccine are viable, giving $3 \times 10^8$ viable organisms in 10 mg moist weight/ml.

*Whole body irradiation*.—Rats were exposed to 450 rad whole body $\gamma$-irradiation from a $^{60}Co$ source at the rate of 7 rad/min, 24 h before use.

*Methods of treatment*.—Normal or whole body irradiated rats were injected s.c. with a mixture of defined numbers of tumour cells ($5 \times 10^5$ to $10^6$) prepared from solid tissue or harvested from *in vitro* culture, and 200 to 500 $\mu$g moist weight of BCG organisms. In some cases, animals received a simultaneous challenge of $10^6$ tumour cells alone at a contralateral subcutaneous site.

*Influence of whole body irradiation on local tumour-suppressive action of BCG*.—Table I shows the tumour-suppressive action of BCG where injected s.c. in admixture with cells of sarcoma Mc7 prepared from solid tissue or harvested from *in vitro* culture into normal rats and animals exposed to 450 rad $\gamma$-irradiation. In all tests, with both normal and irradiated animals, admixture with BCG prevented tumour development in almost all rats, tumour cells alone, whether from solid *in vivo* growths or *in vitro* culture, growing out in the majority of normal or irradiated animals.

*Influence of whole body irradiation on induction of tumour immunity by mixed tumour cell + BCG inocula*.—It has previously been established that injection of a mixed inoculum of sarcoma Mc7 cells and BCG elicits tumour-specific immunity against further challenge with cells of the same tumour (Baldwin and Pimm, 1973). Moreover, treatment of rats with

TABLE I.—*Growth of Sarcoma Mc7 Cells Injected Alone or Mixed with BCG into Normal or Irradiated Rats*

| | Mixed inoculum | | Whole body irradiation* | Tumour |
|---|---|---|---|---|
| Expt. | No. cells | $\mu$g BCG | (rad) | takes |
| 1 | $5\times10^5$ | — | — | 5/5 |
| | $5\times10^5$ | 200 | — | 0/5 |
| | $5\times10^5$ | — | 450 | 5/5 |
| | $5\times10^5$ | 200 | 450 | 0/5 |
| 2 | $10^6$ | — | — | 5/6 |
| | $10^6$ | 200 | — | 0/6 |
| | $10^6$ | — | 450 | 5/5 |
| | $10^6$ | 200 | 450 | 0/4 |
| 3 | $10^6$ | — | — | 4/4 |
| | $10^6$ | 500 | — | 2/5 |
| | $10^6$ | — | 450 | 5/5 |
| | $10^6$ | 500 | 450 | 0/5 |
| 4† | $10^6$ | — | — | 6/6 |
| | $10^6$ | 500 | — | 0/5 |
| | $10^6$ | — | 450 | 5/6 |
| | $10^6$ | 500 | 450 | 0/6 |

* 24 h before tumour injection.
† Sarcoma Mc7 cells from *in vitro* culture.

such mixed inocula can be used for specific active immunotherapy of a distant challenge inoculum of up to $10^6$ sarcoma Mc7 cells. Table II summarizes results of tests to examine the influence of whole body irradiation on the ability of animals rejecting mixed cell + BCG inocula to control the growth of a simultaneous challenge with tumour cells alone. In the first test only 2/7 normal animals rejecting mixed inocula of $10^6$ sarcoma Mc7 cells and 500 $\mu$g moist weight BCG failed to reject a simultaneous challenge inoculum of $10^6$ cells alone on the other side of the body. In contrast, tumours grew out at the challenge site in all (7/7) rats receiving whole body irradiation, even though the animals all rejected the mixed inoculum of tumour cells + BCG. In two further tests, while the challenge inoculum of cells alone grew out in only 4/10 normal animals rejecting cells + BCG on the other side of the body, this immunotherapeutic effect was totally abolished in pre-irradiated animals, challenge inocula growing out in all (13/13) rats.

In the final test, using Mc7 cells harvested from *in vitro* culture, 4/5

TABLE II.—*Growth of* $10^6$ *Sarcoma Mc7 Cells* in Normal or Irradiated Rats Rejecting Mixed Inocula of* $10^6$ *Tumour Cells + 500 μg BCG*

| Expt. | Whole body irradiation (rad) | Takes in Test | Control |
|---|---|---|---|
| 1 | — | 2/7 | 6/6 |
| | 450 | 7/7 | 6/6 |
| 2 | — | 3/6 | 6/6 |
| | 450 | 6/6 | 5/5 |
| 3 | — | 1/4 | 7/7 |
| | 450 | 7/7 | 7/7 |
| 4† | — | 1/5 | 6/6 |
| | 450 | 5/6 | 5/6 |

* Injected s.c. at contralateral site at the same time as the mixed inoculum.
† Sarcoma Mc7 cells from *in vitro* culture.

normal animals rejecting mixed inocula of tumour cells + BCG rejected a contralateral challenge of $10^6$ tissue-culture-derived cells, but this therapeutic response was abrogated in 5/6 pre-irradiated rats.

These studies demonstrate that whole body irradiation (450 rad) 24 h beforehand does not abrogate the local suppressive effect of BCG injected in admixture with sarcoma Mc7 cells. In contrast, the development of tumour-specific host immunity, normally occurring concomitantly with rejection of mixed inocula, was totally abrogated by whole body irradiation. The possibility that local contact suppression mediated by BCG was due to lymphoid cells present in tumour cell suspensions is excluded by the identical results achieved with tissue-culture-derived cells.

These findings are in contrast to those of Bartlett *et al.* (1972) and Chung *et al.* (1973) where full host immunocompetence, or at least prior immunization to BCG, was necessary for local suppression of mouse sarcomata. However, Moore *et al.* (1975), in a series of experiments with transplanted sarcomata in the rat, have shown that immunosuppression by sublethal whole body irradiation, with or without prior thymectomy, did not abrogate the local suppressive action of BCG. In the present studies, the level of immunosuppression produced by simple whole body irradiation has not been characterized, but it has been clearly demonstrated that irradiated animals, while rejecting mixed inocula of cells + BCG, fail to develop the systemic tumour immunity normally capable of controlling a simultaneous challenge with tumour cells alone. The present findings and those of Moore *et al.* (1975) demonstrate, therefore, that fundamentally different host responses are involved in these two events and that full host immunocompetence is not necessary for BCG contact therapy. This is further supported by the previous demonstration of BCG contact suppression of rat tumour xenografts in athymic nude mice (Pimm and Baldwin, 1975).

Although the evidence presented here suggests that augmented systemic responses to tumour-associated rejection antigens are not essential for BCG contact suppression, the nature of the responses involved have yet to be elucidated. It has been demonstrated, however, that with rat tumours, both in syngeneic hosts and athymic mice, the local suppressive action of BCG can be abrogated by silica treatment of the host (Hopper, Pimm and Baldwin, 1976). Silica is known to be selectively toxic for macrophages and the probability that local BCG activation of host macrophages is the primary tumour-suppressive event in adjuvant contact therapy with this agent is under investigation.

This work was supported by the Cancer Research Campaign. BCG vaccine was supplied by Glaxo Research Ltd. We thank Mrs A. P. Wilcox for skilful technical assistance.

REFERENCES

BALDWIN, R. W. & PIMM, M. V. (1973) BCG Immunotherapy of a Rat Sarcoma. *Br. J. Cancer*, **28**, 281.

BARTLETT, G. L., ZBAR, B. & RAPP, H. J. (1972) Suppression of Murine Tumor Growth by Immune Reaction to the Bacillus Calmette Guérin Strain of Mycobacterium Bovis. *J. natn. Cancer Inst.*, **48**, 245.

CHUNG, E. B., ZBAR, B. & RAPP, H. J. (1973) Tumor Regression Mediated by *Mycobacterium bovis* (Strain BCG). Effects of Isonicotinic Acid Hydrazide, Cortisone Acetate, and Antithymocyte Serum. *J. natn. Cancer Inst.*, **51**, 241.

HOPPER, D. G., PIMM, M. V. & BALDWIN, R. W. (1976) Silica Abrogation of Mycobacterial Adjuvant Contact Suppression of Tumour Growth in Rats and Athymic Mice. *Cancer Immunology & Immunotherapy*, (in the press).

LAUCIUS, J. F., BODURTHA, A. J., MASTRANGELO, M. J. & CREECH, R. M. (1974) Bacillus Calmette-Guérin in the Treatment of Neoplastic Disease. *J. Reticuloendothel. Soc.*, **16**, 347.

MOORE, M., LAWRENCE, N. & NISBET, N. W. (1975) Tumour Inhibition Mediated by BCG in Immunosuppressed Rats. *Int. J. Cancer*, **15**, 897.

PIMM, M. V. & BALDWIN, R. W. (1975) BCG Immunotherapy of Rat Tumours in Athymic Nude Mice. *Nature, Lond.*, **245**, 77.